# Difficulties of Preoperative Diagnosis of Cribriform Morular Thyroid Carcinoma

**DOI:** 10.1155/2024/6517236

**Published:** 2024-05-28

**Authors:** Li-Hsin Pan, Jen-Fan Hang, Jui-Yu Chen, Po-Sheng Lee, Yun-Kai Yeh, Tai-Jung Huang, Chii-Min Hwu, Chin-Sung Kuo

**Affiliations:** ^1^Section of Endocrinology and Metabolism, Department of Medicine, Taipei City Hospital Zhongxing Branch, Taipei, Taiwan; ^2^School of Medicine, National Yang Ming Chiao Tung University, Taipei, Taiwan; ^3^Department of Pathology and Laboratory Medicine, Taipei Veterans General Hospital, Taipei, Taiwan; ^4^Division of General Surgery, Department of Surgery, Taipei Veterans General Hospital, Taipei, Taiwan; ^5^Institute of Biology and Anatomy, National Defense Medical Center, Taipei, Taiwan; ^6^Department of Medicine, Taipei Veterans General Hospital, Taipei, Taiwan; ^7^Section of Endocrinology and Metabolism, Department of Medicine, Taipei Veterans General Hospital, Taipei, Taiwan; ^8^Department of Medical Education, Taipei Veterans General Hospital, Taipei, Taiwan

## Abstract

**Background:**

Cribriform morular thyroid carcinoma has been recently renamed in the 2022 WHO classification as a thyroid tumor of uncertain histogenesis. The epidemiologic, pathological, and pathophysiological characteristics distinguish it from papillary thyroid carcinoma (PTC). Preoperative genetic testing plays a role in facilitating the differential diagnosis.

**Methods:**

This report presents a confirmed case of cribriform morular thyroid carcinoma. Initially, fine-needle aspiration cytology suggested a diagnosis of PTC. However, a genetic analysis did not reveal the typical mutations associated with follicular-cell-derived neoplasms.

**Results:**

A 31-year-old woman was found to have a thyroid nodule at the left lobe measuring 11.8 × 10.2 × 12.4 mm. Ultrasonography indicated a hypoechoic, solid nodule with regular margins. Cytology revealed a papillary structure of tall cells, leading to a PTC diagnosis. Nevertheless, the genetic analysis failed to detect mutations such as *BRAF V600E*, *NRAS Q61R*, *NRAS Q61K*, *HRAS Q61R*, *or HRAS Q61K* mutation or the fusion of *CCDC6-RET*, *NCOA4-RET*, *PAX8-PPARG*, *ETV6-NTRK3*, *TPM3-NTRK1*, *IRF2BP2-NTRK1*, or *SQSTM1-NTRK1* in the aspirated follicular cells. The patient subsequently underwent total thyroidectomy with central lymph node dissection. Pathological examination revealed a cribriform pattern of spindle-shaped cells with morular areas. Immunohistochemical staining showed positive results for *β*-catenin and TTF-1, except in the morular regions, and negative results for PAX8, thyroglobulin, and BRAF (clone VE1). The diagnosis was confirmed to be cribriform morular thyroid carcinoma.

**Conclusion:**

Significant cytological similarity exists between PTC and cribriform morular thyroid carcinoma. Preoperative genetic analysis is important to differentiate these two diseases. Cribriform morular thyroid carcinoma can be differentiated from common follicular-cell-derived tumors by the absence of typical mutations; the presence of nuclear and cytoplasmic expressions of *β*-catenin; the presence of TTF-1, except in morular areas; and the absence of thyroglobulin.

## 1. Introduction

In 2022, the World Health Organization (WHO) updated the pathologic classification of thyroid neoplasms. In this new classification, cribriform morular thyroid carcinoma has been designated as thyroid tumors of uncertain histogenesis [1]. Previously, this tumor has been considered a variant of papillary thyroid cancer (PTC). However, the absence of common mutations associated with thyroid follicular cells and the lack of expression for thyroglobulin distinguish this disease from usual PTC. The pathogenesis of cribriform morular thyroid cancer involves the Wnt/*β*-catenin pathway, often associated with potential germline or sporadic *APC* mutations, and/or with functionally equivalent genes such as *CTNNB1* [2, 3] and *AXIN1* [4].

Cribriform morular thyroid carcinoma is a relatively rare form of thyroid cancer, and it is more commonly reported in East Asian populations. Its prevalence is approximately 1.4% based on data from a case series in Taiwan [5]. The age of onset for this type of cancer tends to be comparatively lower, with the peak incidence occurring in the third decade of life (mean age: 26 years), which contrasts with PTC [4, 6]. Additionally, a female predominance was observed, with a female-to-male ratio of approximately 61 : 1 [4] and with an unclear association of radiation exposure [7]. In cytological findings, a significant similarity existed between PTC and cribriform thyroid carcinoma such as the presence of ground-glass nuclei and distinctive nuclear clearing or a papillary architecture featuring columnar or spindle cells [8].

It would be challenging to make a preoperative diagnosis of cribriform morular thyroid carcinoma. We describe a case of cribriform morular thyroid carcinoma presented as the fine-needle cytological diagnosis of PTC with absence of preoperative genetic testing of common mutations or fusions.

## 2. Case Presentation

**Figure 1:**
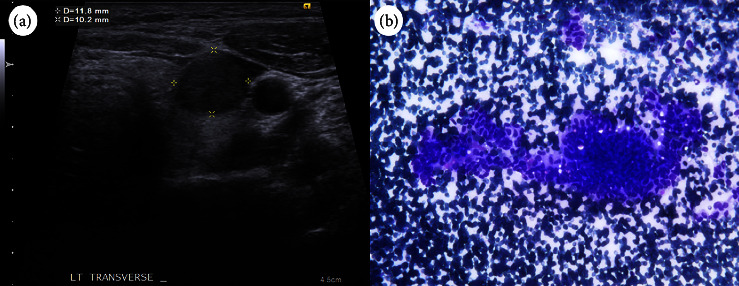
(a) Thyroid ultrasonography images depicting a solid, hypoechoic nodule with a well-defined border on the left lobe. (b) Fine-needle aspiration cytology findings revealing numerous spindle-shaped tall cells with a densely packed structure and an enlarged nucleus-to-cytoplasm ratio.

**Figure 2:**
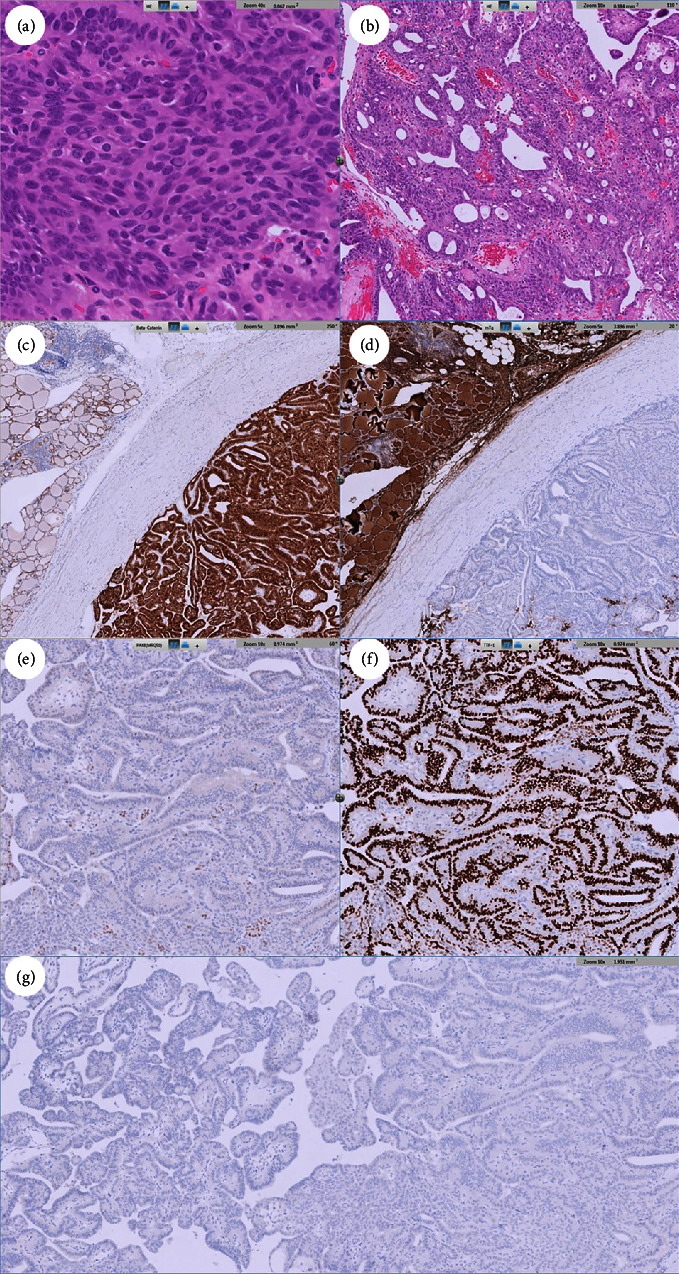
(a) Microscopic view of the tumor, revealing numerous spindle-shaped cells with a morular arrangement. (b) Cribriform structure was prominent. (c) The tumor was positive for *β*-catenin in the cytoplasm and nuclei and (d) negative for thyroglobulin. (e) The tumor stained negative for PAX8 but (f) positive for TTF-1 in areas other than morular areas. (g) Staining for BRAF VE1 was negative.

Our case is of a 31-year-old woman who had no underlying disease. She discovered a thyroid nodule during a routine health examination and presented at our hospital for further investigation. She did not have history of radiation exposure. She had no history of colonic polyps or a family history of familial adenomatous polyposis (FAP) or thyroid cancer. She worked as an office lady. She never smoked, consumed alcohol, chewed betel nuts, or used any illegal substances. Laboratory tests indicated normal levels of thyroid-stimulating hormone and free T4, but the anti-thyroid-peroxidase antibody levels were higher than average (548 IU/mL, reference range: <34 IU/mL). Thyroid ultrasonography revealed a heterogeneous echo texture in the thyroid gland and a solid, hypoechoic, wider-than-tall nodule measuring 11.8 × 10.2 × 12.4 mm in size with a smooth margin and no calcification (Figure 1(a)). According to the hypoechoic and solid features mentioned above, the Thyroid Imaging Reporting and Data System score was 4. Fine-needle aspiration cytology revealed papillary growth of tall and spindle-shaped cells with elongated nuclei and an increased nucleus-to-cytoplasm ratio (Figure 1(b)). The cytology was classified under Bethesda category VI, and the diagnosis was PTC. To screen for common genetic alterations, we employed the ThyroSCAN Cancer Diagnostics Kit (Quak BioTechnology). The analysis of the targeted genes did not reveal the presence of mutations such as *BRAF V600E*, *NRAS Q61R*, *NRAS Q61K*, *HRAS Q61R*, or *HRAS Q61K* mutations nor fusions of *CCDC6-RET*, *NCOA4-RET*, *PAX8-PPARG*, *ETV6-NTRK3*, *TPM3-NTRK1*, *IRF2BP2-NTRK1*, or *SQSTM1-NTRK1* in the aspirated samples. In our differential diagnosis, we considered cribriform morular thyroid cancer and the tall cell variant of PTC. No abnormal lymph node was observed in thyroid ultrasonography or neck computed tomography findings. Subsequently, the patient underwent total thyroidectomy and central lymph node dissection. A pathological examination revealed a mixed pattern consisting of cribriform and morular features in the stratified tall cells, along with evidence of angioinvasion and capsular invasion. Immunohistochemical staining indicated a positive result for *β*-catenin (cytoplasmic and nuclear expression) but negative results for thyroglobulin, PAX8, and BRAF (clone VE1). The result for TTF-1 was also positive, except in the morular regions (Figure 2). The final diagnosis was cribriform morular thyroid carcinoma measuring 1.2 cm in the maximal length without extrathyroidal extension, lymph node invasion, or distant metastasis, and it was categorized as stage I, pT1bN0M0. The patient had a smooth postoperative recovery and was discharged without any complications.

## 3. Discussion

This case report proposes the diagnostic value of preoperative genetic testing with all-negative results in thyroid nodules classified as malignant (Bethesda VI)—PTC. A recent mutational study of PTCs conducted by targeted RNA-based next-generation sequencing using a FusionPlex Pan Solid Tumor v2 panel in Taiwan identified 86.8% (481/554) incidence of *BRAF V600E* mutation, 1.4% (8/554) of cribriform morular thyroid carcinoma, 11.2% (62/554) *BRAF-negative* PTCs that are mostly included in the 12-panel mutation of ThyroSCAN except for 5 cases of *BRAF* fusions, 2 cases of *KRAS Q61K*, 1 case of *ALK* fusion, 1 case of *FGFR1* fusion, and 9 cases of not detected mutation [5]. Therefore, cribriform morular thyroid carcinoma would be considered as potential diagnosis in fine-needle cytology class VI with all-negative results of ThyroSCAN genetic testing of thyroid nodules.

Molecular testing may be adopted to determine the malignancy risk associated with thyroid nodules. Among these tests, the detection of *BRAF V600E* mutation is highly specific for identifying PTC. When combined with thyroid ultrasonography or cytology, *BRAF V600E* detection significantly enhances the diagnostic sensitivity for PTC [9, 10]. Preoperative use of molecular analyses has been demonstrated to accurately identify most papillary cell lesions, medullary thyroid cancer, and parathyroid tumors [11]. This information can be invaluable in planning the extent of surgical interventions particularly for nodules classified as Bethesda category V and VI [12, 13]. Nevertheless, molecular testing is still difficult to distinguish benignity from malignancy in follicular and oncocytic lesions [11].

In terms of pathological features, cribriform morular thyroid carcinoma typically presents grossly with encapsulation and the formation of divided lobules inside. Microscopically, various growth patterns may be observed, including papillary, follicular, or cribriform ones. The tumor cells are typically cuboidal, oval, or tall and exhibit prominent cytoplasm, hyperchromatic nuclei, and pseudostratification. At times, the tumor cells aggregate and form squamoid morules. The squamoid morules may have nuclear clearing without features of PTC [14]. Invasion into blood vessels and the capsule has been occasionally reported. The presence of psammoma bodies is rare [4]. The Ki-67 index that is not required for diagnosis of cribriform morular thyroid cancer may show high despite excellent prognosis [4, 15]. Regarding immunohistochemistry, *β*-catenin expression is prominent in the nuclei or cytoplasm of cells in cribriform morular thyroid carcinoma, whereas in PTC, *β*-catenin is mainly expressed on plasma membranes [3, 7]. Furthermore, morular areas of cribriform morular thyroid carcinoma often lack TTF-1 expression but have CDX2, CD5, and CK5. The cribriform regions also exhibit aberrant progesterone and estrogen receptor expression [1]. On the other hand, PAX8 expression may not be negative. Some case reports revealed positive PAX8 in immunohistochemistry of the tumor cells [14, 16]. Additionally, more than 30% of cribriform morular thyroid carcinoma tumors harbor somatic *APC* mutations, whereas up to 50% of cancer cells harboring somatic *APC* mutations can be found in patients with germline *APC* aberrations. In terms of the underlying mechanisms, the APC protein is associated with glycogen synthase kinase 3*β* (GSK3*β*), AXIN, and casein kinase 1*α* (CK1*α*). This complex plays a crucial role in tightly regulating the cytoplasmic levels of *β*-catenin. Mutations in the *APC* gene lead to the formation of a truncated APC protein that lacks the binding site for *β*-catenin. Consequently, *β*-catenin is unable to undergo proteasomal degradation. The stabilized *β*-catenin is then translocated into the cell nuclei, where it binds to LEF/TCF proteins. This interaction results in the constitutive expression of MYC, CCND1 (cyclin D1), Axin2, and DKK1 and leads to increased cell proliferation and dedifferentiation [4]. Echegoyen-Silanes et al. also identified two somatic mutations (c.3428_3429insA, p. (Tyr1143Ter) and c.3565del, p. (Ser1189Hisfs*∗*76) of *APC* in a 29-year-old female patient. They postulated that cribriform morular thyroid carcinoma might stem from endodermal progenitors, exhibiting an intestine-like phenotype due to prematurely terminated cell differentiation. [16]. Moreover, in cribriform morular thyroid carcinoma, other genetic alterations have been reported, including *RET/PTC* rearrangements, *PIK3CA* mutations, and *RAS* gene mutations. These genetic alterations can function as upstream effectors in the WNT/*β*-catenin pathway [17].

The clinical features of cribriform morular thyroid carcinoma may be associated with FAP due to *APC* mutations. In a Japanese case series, approximately 39% of patients with cribriform morular thyroid carcinoma were found to have FAP. These patients with FAP tended to present with multiple thyroid tumors compared with those with the sporadic form of the disease. Among the patients with FAP, patients who initially had thyroid tumors tended to have larger tumors compared with those who initially presented with colonic polyps. Moreover, the clinical course of cribriform morular thyroid carcinoma is generally indolent, with a low mortality rate [18]. Lymph node extension occurs in only approximately 10% of patients, and distant metastases are uncommon (6%), typically occurring in older patients [4, 6]. When distant metastases do occur, they often involve the lungs, bones, and brain [4]. In terms of the ultrasonic findings of cribriform morular thyroid carcinoma, the lesions are frequently described as solid, hypoechoic, oval to circular, heterogeneous, and circumscribed, typically lacking calcifications or a hypoechoic halo [19]. Management of cribriform morular thyroid carcinoma typically follows a similar approach to that of PTC. Total thyroidectomy is usually considered sufficient for effective disease control, with or without lymph node dissection. In cases of local invasion, external beam radiotherapy can be considered as a treatment option. Furthermore, screenings for colonic polyps and congenital hypertrophy of the retinal pigment epithelium must be performed to exclude germline *APC* mutations and association with FAP [6, 20]. The overall prognosis for individuals with cribriform morular thyroid carcinoma is generally favorable. The reported recurrence rate is approximately 8.5%, which is lower than the 16.1% recurrence rate observed in classical PTC [21]. Perrier et al. found that the 5-year and 20-year survival rates for 11 patients suspected to have cribriform morular thyroid carcinoma were 90% and 77%, respectively [22]. The disease-related mortality in these cases was 2%, which is similar to the 2.5% reported in classical PTC cases [6].  Table 1 summarizes the differences in several clinical and pathological features between classical PTC and cribriform morular thyroid carcinoma.

In conclusion, cribriform morular thyroid carcinoma was seldom reported in Taiwan. Making the correct preoperative diagnosis is difficult based on fine-needle aspiration cytology. This case underscores the importance of preoperative molecular analysis in achieving an accurate diagnosis. The definitive diagnosis, however, is histopathological, based on the recognition of its peculiar tumor growth pattern together with nuclear and cytoplasmic positivity for beta-catenin.

## Figures and Tables

**Table 1:** Differences between classic papillary thyroid cancer and cribriform morular thyroid carcinoma [1, 2, 4, 5, 22].

Characteristics	Classical papillary thyroid cancer	Cribriform morular thyroid carcinoma
Mean age	45	26
Female-to-male ratio	3 : 1	61 : 1
Lymph node invasion	43%	∼10%
Cancer recurrence	16.1%	8.5%
Mortality	2.5%	2%
Psammoma body	May be present	Rarely present
Thyroglobulin expression	Positive	Negative
TTF-1 expression	Positive	Positive in cribriform areas
*β*-catenin expression	Plasma membrane	Cytoplasm and nuclei
*BRAF* mutation	May be positive	Negative
*APC* mutation	Negative	May be positive
Tumorigenic pathway	MAPK pathway	Wnt/*β*-catenin pathway

## Data Availability

The data used to support the findings of this study are available from the corresponding authors upon reasonable request.

## References

[1] Baloch Z. W., Asa S. L., Barletta J. A. (2022). Overview of the 2022 WHO classification of thyroid neoplasms. *Endocrine Pathology*.

[2] Xu B., Yoshimoto K., Miyauchi A. (2003). Cribriform‐morular variant of papillary thyroid carcinoma: a pathological and molecular genetic study with evidence of frequent somatic mutations in exon 3 of the *β*‐catenin gene. *The Journal of Pathology*.

[3] Boyraz B., Sadow P. M., Asa S. L., Dias-Santagata D., Nosé V., Mete O. (2021). Cribriform-morular thyroid carcinoma is a distinct thyroid malignancy of uncertain cytogenesis. *Endocrine Pathology*.

[4] Cameselle-Teijeiro J. M., Peteiro-González D., Caneiro-Gómez J. (2018). Cribriform-morular variant of thyroid carcinoma: a neoplasm with distinctive phenotype associated with the activation of the WNT/*β*-catenin pathway. *Modern Pathology*.

[5] Hang J. F., Chen J. Y., Kuo P. C. (2023). A shift in molecular drivers of papillary thyroid carcinoma following the 2017 World health organization classification: characterization of 554 consecutive tumors with emphasis on BRAF-negative cases. *Modern Pathology*.

[6] Lam A. K., Saremi N. (2017). Cribriform-morular variant of papillary thyroid carcinoma: a distinctive type of thyroid cancer. *Endocrine-Related Cancer*.

[7] Or Koca A., Güler Şimşek G. (2023). Post-radiotherapy cribriform-morular thyroid carcinoma. *Journal of Clinical Laboratory Analysis*.

[8] Hirokawa M., Maekawa M., Kuma S., Miyauchi A. (2010). Cribriform-morular variant of papillary thyroid carcinoma--cytological and immunocytochemical findings of 18 cases. *Diagnostic Cytopathology*.

[9] Antonia T. D., Maria L. I., Ancuta-Augustina G. G. (2023). Preoperative evaluation of thyroid nodules-Diagnosis and management strategies. *Pathology, Research and Practice*.

[10] Adeniran A. J., Theoharis C., Hui P. (2011). Reflex BRAF testing in thyroid fine-needle aspiration biopsy with equivocal and positive interpretation: a prospective study. *Thyroid*.

[11] Nikiforova M. N., Mercurio S., Wald A. I. (2018). Analytical performance of the ThyroSeq v3 genomic classifier for cancer diagnosis in thyroid nodules. *Cancer*.

[12] Schumm M. A., Shu M. L., Hughes E. G. (2023). Prognostic value of preoperative molecular testing and implications for initial surgical management in thyroid nodules harboring suspected (Bethesda V) or known (Bethesda VI) papillary thyroid cancer. *JAMA Otolaryngol Head Neck Surg*.

[13] Sipos J. A., Ringel M. D. (2023). Molecular testing in thyroid cancer diagnosis and management. *Best Practice and Research Clinical Endocrinology and Metabolism*.

[14] Sahu A., Shahin M., Jain P., Sultania M., Ayyanar P. (2023). Cribriform morular thyroid carcinoma: a rare case and associated uncommon features. *International Journal of Surgical Pathology*.

[15] Cameselle-Teijeiro J., Menasce L. P., Yap B. K. (2009). Cribriform-morular variant of papillary thyroid carcinoma: molecular characterization of a case with neuroendocrine differentiation and aggressive behavior. *American Journal of Clinical Pathology*.

[16] Echegoyen-Silanes A., Pineda-Arribas J. J., Sánchez-Ares M. (2023). Cribriform morular thyroid carcinoma: a case report with pathological, immunohistochemical, and molecular findings suggesting an origin from follicular cells (or their endodermal precursors). *Virchows Archiv*.

[17] Abbosh P. H., Nephew K. P. (2005). Multiple signaling pathways converge on beta-catenin in thyroid cancer. *Thyroid*.

[18] Ito Y., Miyauchi A., Ishikawa H. (2011). Our experience of treatment of cribriform morular variant of papillary thyroid carcinoma; difference in clinicopathological features of FAP-associated and sporadic patients. *Endocrine Journal*.

[19] Chong Y., Shin J. H., Oh Y. L., Han B. K., Ko E. Y. (2013). Cribriform-morular variant of papillary thyroid carcinoma: ultrasonographic and clinical characteristics. *Thyroid*.

[20] Chen C. S., Phillips K. D., Grist S. (2006). Congenital hypertrophy of the retinal pigment epithelium (CHRPE) in familial colorectal cancer. *Familial Cancer*.

[21] Xing M., Alzahrani A. S., Carson K. A. (2015). Association between BRAF V600E mutation and recurrence of papillary thyroid cancer. *Journal of Clinical Oncology*.

[22] Perrier N. D., van Heerden J. A., Goellner J. R. (1998). Thyroid cancer in patients with familial adenomatous polyposis. *World Journal of Surgery*.

